# Complete mitochondrial genome of *Leptodius sanguineus* (Decapoda, Xanthidae)

**DOI:** 10.1080/23802359.2016.1192505

**Published:** 2016-07-12

**Authors:** Jin-Mo Sung, JeaHyun Lee, Seong-Geun Kim, Mustafa Zafer Karagozlu, Chang-Bae Kim

**Affiliations:** a Department of Life Science, Sangmyung University, Seoul, Korea;; b Bionics Co, Ltd, Seoul, Korea

**Keywords:** Arthropoda, complete mitochondrial genome, decapoda, *Leptodius sanguineus*, xanthidae

## Abstract

The complete mitochondrial genome sequenced from a xanthid crab, *Leptodius sanguineus* which was collected from a rocky intertidal area in Chuuk lagoon. The mitochondrial genome size of *L. sanguineus* evaluated as 15,480 bp with 33.6% A, 24% C, 11.2% G and 31.2% T and mitochondrial gene order of *L. sanguineus* is typical to brachyuran species. Phylogenetic analysis shows that the family Xanthidae has sister group relationship with a lineage including the families Portunidae and Menippidae in the subsection Heterotremata. This is the first report for the complete mitochondrial genome from the xanthid crabs.

Xanthidae is one of the largest crab family which includes 572 species in 133 genera (De Grave et al. 2009). In spite of the species richness, there is no record of the complete mitochondrial genome from the family Xanthidae. In this study, we evaluate the complete mitochondrial genome of *Leptodius sanguineus*.

The species have been collected from the rocky intertidal zone of Weno Island, Chook Lagoon, Federated States of Micronesia (7°27′48″N, 151°52′39″E) on 26 February 2015 and preserved in 97% ethanol and the specimen deposited in the Marine Biodiversity Institute of Korea (MABIK CR00235263).

Mitochondrial genome length of *L. sanguineus* is 15,480 bp (GenBank accession number KT896744). The mitochondrial genome is composed of 13 protein coding genes (PCGs), 2 ribosomal RNA genes and 22 tRNA genes, and mitochondrial gene order of *L. sanguineus* is typical to brachyuran species (Tan et al. 2014). There are eight overlapping regions with 1–7 bp in length. The genome has 13 intergenic sequences varying from 1 to 47 bp in length. The largest intergenic sequence is located between NAD5 and NAD4. The lengths of 12S rRNA and 16S rRNA are 829 and 1336 bp. Protein-coding genes use three initiation codons (ATG, AAT and ATT). There are three termination codons (TAA, TAG and T––). The nucleotide distribution of the mitochondrial genome is 33.6% A, 24% C, 11.2% G and 31.2% T. There is a putative control region which is 594 bp between 12S rRNA and t-RNA-Ile. There are several common microsatellites in a putative control region such as TATATA, ATATAT and AAATAA. The most common motif is TA–, which is seen 192 times in the putative control region. The phylogenetic study showed that *L. sanguineus* positioned in the family Xanthidae and it has sister group relationship with a lineage including the families Portunidae and Menippidae in the subsection Heterotremata (Figure 1). Similar results showed previously by the combination of the six nuclear genes- and two mitochondrial genes-based molecular studies (Tsang et al. 2014). This is the first report for *L. sanguineus* complete mitochondrion sequence information. This mitochondrial genome gives genetic markers for phylogenetics of the xanthid crabs which will be a part of mitochondrial genome library to provide evolutionary and systematic studies.

**Figure 1.**
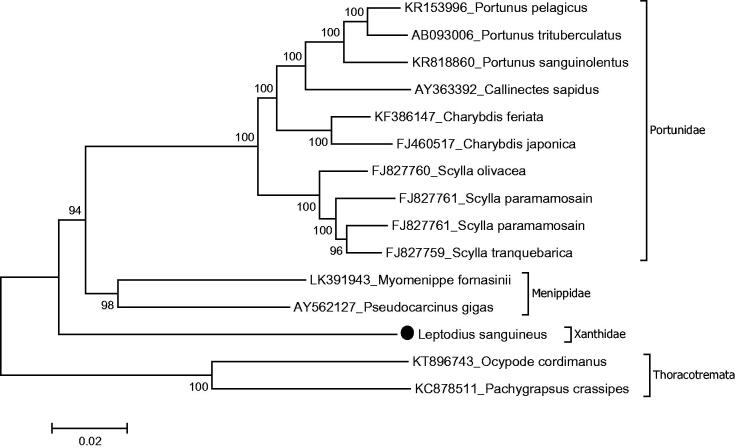
Phylogenetic relationships of *L. sanguineus* in the subsection Heterotremata due to amino acid sequences of all protein-coding genes. Purified libraries were profiled using the bioanalyzer (Agilent, CA, USA) and sequenced with the Illumina MiSeq platform to yield 300 bp paired end reads. Mitochondrial genes were assembled and annotated by MITObim software (Hull, UK) (Hahn et al. 2013) and MITOS web server (Leipzig, Germany) (Bernt et al. 2013) and the annotation of mitochondrial genome sequences was refined using Geneious software version 9.1.3 ((Geneious, Auckland, New Zealand), Kearse et al. 2012). The phylogeny of *L. sanguineus* was reconstructed with maximum-likelihood statistical method by Mega 7 software verwsion 7.0.14 (MEGA, PA, USA) (Kumar et al. 2016). mtREV with Freqs (+F) model used for amino acid substitution and bootstrap method replicated 1000 times for the statistical test of branches of phylogeny. Complete mitogenomes were retrieved from the GenBank to reconstruction of the phylogenetic tree of the Heterotomata. Two species belong to the subsection Thoracotremata chosen as outgroup.
